# Supplementary material for: Are carbon emissions trading and green financial instruments synergistic? -Comprehensive quantitative research based on content analysis
Source: PLoS One. 2024 Mar 7;19(3):e0298601. doi: 10.1371/journal.pone.0298601 (PMC10919601; doi:10.1371/journal.pone.0298601)
Supplement: S1 Data — (ZIP) [file pone.0298601.s001.zip › Data/地级市Z_F工作报告.zip!/╔╜╬≈/┴┘╖┌╩╨/└┤╫╘╣1⁄2╓┌║┼í╛┬φ┐╦╩2╛▌═°í┐_2011.docx]

各位代表：

　　现在，我代表市人民政府，向大会作政府工作报告，请予审议，并请各位政协委员和其他列席人员提出意见。

一、2010年工作回顾

　　2010年是“十一五”收官之年，也是我们砥砺奋进、取得丰硕成果的一年。一年来，面对复杂多变的市场形势和激烈区域竞争，面对爬坡过坎、负重赶超的严峻挑战，在省委、省政府和市委的正确领导下，在市人大和市政协的监督支持下，市政府团结带领全市人民，深入贯彻落实科学发展观，紧紧围绕“转型跨越、先行发展”的实践主题，突出“打牢基础、调整结构、转变方式、促进发展、惠及民生”的工作重点，开拓创新，奋力拼搏，胜利完成了市二届人大六次会议确定的各项目标任务，谱写了全市经济社会发展的新篇章。

——克服不利因素叠加影响，经济实现平稳较快增长。我们积极应对市场需求不足、主导性企业特别是煤焦企业开工率较低、节能减排任务加大、国家货币政策趋紧等诸多困难，及时采取有效措施，不断强化调控职能，保障经济顺畅运行，主要经济指标实现两位数增长，增速均居全省前列。全市生产总值完成892.1亿元，同比增长16.9%；规模以上工业增加值完成465.1亿元，增长30.1%；社会消费品零售总额完成306.5亿元，增长18.7%；海关进出口总额完成6.5亿美元，增长83.3%；财政总收入完成160.3亿元，增长16.4%；一般预算收入完成75.4亿元，增长19.9%；城镇居民人均可支配收入达到13831元，增长12.9%；农民人均纯收入达到5287元，增长11.3%。特别是全市人均生产总值首次突破3000美元大关，标志着我市经济社会发展进入了新阶段。

——项目建设力度空前，基础设施显著改善。继续开展“项目建设年”活动，启动实施216项市级重点工程，完成固定资产投资545.58亿元，同比增长30.2%，投资总量和增幅居全省三甲。特别是在交通基础设施方面，我们打了一个翻身仗。备受社会关注的乔李民航机场复航改造项目获国家审批，机场大道完成路基工程；临大一级公路、沿黄干线、209、309等国省道主干线改造全面完成，改变了西山地区交通不畅的状况。晋侯高速全线开通，市区城西一级客运站、临吉高速、祁临高速临汾北环、京昆与青兰高速临汾联络线进展顺利，霍永高速已经开工。中南部铁路通道、大西高铁客运专线、张台铁路、南同蒲电气化改造、侯西复线扩能等五条铁路加快推进。县乡村三级联网及农村公路完成3114公里，实现了村村通油（水泥）路全覆盖。建设500千伏变电站1座，220千伏变电站7座，110千伏变电站16座，形成了500千伏纵贯南北、220千伏环网运行、110千伏分区供电的格局。

——开拓思路创新方法，破解发展难题闯出新路。围绕解决资金、土地两大难题，采取“争、贷、引、融”等方法，筹集资金330亿元，支持项目建设。其中，向上级申报项目1137项，争取资金17.59亿元；深化银企合作，全年新增贷款102.9亿元，同比增长23.9%，贷款余额达到536亿元；强化融资平台建设，成功发行地方债券20亿元；紧盯大企业大财团，全力承接发达地区产业转移，签订项目68个，签约资金2222.7亿元，到位外来投资172.45亿元，签约资金居全省之首，到位资金占年度项目总投资的三分之一。特别是加强与开行合作，争取贷款14.5亿元，支持新高中、新医院等重大项目建设，达成五年投资500亿元协议，建设“百公里汾河新型工业城镇走廊”。与中煤、保利集团签订协议资金1710亿元，改造提升煤化、焦化产业；与新兴际华集团签订协议资金26亿元，发展精密铸造；与上海云峰集团签订协议资金23亿元，开发广胜寺旅游，有效解决了项目融资难的问题。采取“争政策、争指标、严管理”的用地措施，争取国家级复垦整合试点市政策，在全国率先出台了《建设用地复垦整合办法》；争取省追加用地指标7900亩，全年供应建设用地2.88万亩，占全省的31%。

——结构调整取得突破，多元化支柱产业加速生成。坚持以煤为基、多元发展，大力推进产业结构调整。传统产业加速升级。煤炭资源整合和煤矿兼并重组圆满完成。全市煤矿总数压减到129座，经营主体减少到29个，产能提高到1.07亿吨，单井生产能力平均达到83.3万吨，提高了3.62倍，煤炭行业走上了规模化、机械化、清洁化、高效化的轨道。焦化行业两大基地和4个焦化集中区整合重组迈出实质性步伐，新组建了同世达煤化集团和三维瑞德焦化公司。洪洞赵城煤焦化深加工园区被确定为国家级新型工业化产业示范基地。翼钢扩建进展顺利，太钢重组中宇、临钢退城搬迁有序推进。曲沃冶金工业园区被确定为省级新型工业化产业示范基地。霍州兆光2×60万千瓦、大唐国际2×30万千瓦一期建成投产，侯马电厂、临汾中电投两个2×30万千瓦以及霍州国电2×60万千瓦发电项目进展顺利，全市新增电力装机容量90万千瓦，总装机容量达到330万千瓦。新兴产业异军突起。华翔美的35万吨精密铸件一期、华强钢铁20万吨铸件一期、平阳重工液压支架等一批装备制造业项目顺利建成；塔尔纳米保温材料、浮山满星硅酸钙板、晋盛微晶玻璃等新材料项目竣工投产；尧都光宇LED、洪洞双银电热膜、正和超导暖气片、飞虹微纳米光电等高新技术项目势头强劲。特别是与深圳电子商会合作，致力打造侯马开发区电子信息产业园，部分企业已经入驻。第三产业蓬勃发展，出台了《关于加快旅游产业发展的意见》，重点实施22个旅游建设项目，旅游综合收入达到97.27亿元，增长22%。方略保税物流中心已有50余家大型企业入驻，达到一级口岸标准。

——“三农”工作扎实推进，农业基础地位更加稳固。认真落实各项惠农政策，全市财政用于“三农”的支出达到13亿元。农业基础设施建设进一步加强。引沁入汾和川取水输水工程全面竣工，五马水库大坝封顶，汾西灌区节水续建配套工程完成年度任务。治理水土流失面积51.75万亩，新增灌溉面积6.64万亩。全省节水灌溉、水土保持精品工程建设和扶贫工作现场会先后在我市召开。全年粮食总产达到20.74亿公斤，创历史新高。特色高效农业迈出新步伐。“四个百万亩”基地建设快速推进，涌现出尧王台现代农业，曲沃、浮山设施蔬菜等一批先进典型；“393”龙头企业培育工程加快实施，新增农业产业化龙头企业14户，发展农民专业合作社1353个。“五个全覆盖”任务圆满完成。“四化四改”和“六个一”工程深入推进，我市新农村连片推进经验在全省推广。

——城市框架迅速拉大，品位功能大幅提升。《临汾市城市总体规划（2009—2020）》修编已经省政府批复，建城区面积由54平方公里扩大到155平方公里。以西南环路桥工程建成为标志，52.6公里的市区一级环路全线贯通。滨河西路北延、车站街、迎春北街、向阳路、五一东路东段等街道改造工程全部完工。古城公园向市民开放。大唐国际和中电投热源厂、土门水源地和饮用水处理工程建成投入运营，集中供热、供气、供水覆盖面不断扩大。市区垃圾中转站、第二污水处理厂、医疗垃圾处理场投入使用，公厕建设荣获“中国人居环境示范奖”，市容环境进一步改善。特别是投资11亿元，完成汾河城区段生态治理修复一期工程，修复治理面积10.6平方公里，人均城市绿地增加15平方米，市区绿化覆盖率由31.4%增加到42.3%，成为全省面积最大、标准最高的城市生态公园。各县（市、区）污水处理厂全部建成投入使用，垃圾处理场完成年度建设目标。

——节能减排成效明显，环境质量持续改善。认真落实国家节能减排政策，对120户重点耗能企业进行专项监察，推广节能技术20项，完成节能项目112个，取缔汾河沿岸污染企业156家。万元GDP综合能耗3.13吨标煤，比上年下降9.1%；化学需氧量净减排1700吨，下降4.86%；二氧化硫净减排2250吨，下降2.34%，圆满完成了省政府下达的节能减排任务。市区二级以上天数达到338天，同比增加4天，其中一级天数达99天，同比增加31天，综合污染指数为1.71，同比下降0.6%。完成造林绿化63.14万亩，新增造林面积居全省第一，全市森林覆盖率达到28.9％，直接拉动全省森林覆盖率提高1个百分点。

　　——安全工作扎实有效，安全形势明显好转。认真落实企业的安全生产主体责任，切实加强重点领域和关键环节的安全监管，对所有煤矿派驻了“五人监管”小组，对所有非煤矿山及其它工业企业配备了“五大员”。严格落实安全生产十项制度，新出台《安全生产承诺制度》和《一般事故查处挂牌督办办法》。扎实开展隐患排查和专项治理，“春雷”、“利剑”、“铁腕打非”行动和“安全生产百日大会战”成效明显。亿元GDP生产安全事故死亡率下降68%，工矿商贸十万就业人员事故死亡率下降80%，道路交通万车死亡率下降58%，煤炭百万吨死亡率下降81%，全年未发生一次死亡10人以上安全事故，基本扭转了被动局面，安全生产形势明显好转。

——各项改革不断深化，发展活力进一步增强。市县乡三级政府机构改革全面完成。事业单位分类改革稳步推进。5户市属国有企业改革完成破产终结。农村中小金融机构股份制改革实现重大突破，山西尧都农村商业银行正式挂牌，成为我省第一家开业的农村商业银行。医药卫生体制改革取得阶段性成果，11个县（市、区）实施国家基本药物制度。集体林权制度改革全面铺开，完成确权面积891万亩，超省定任务1.17倍，综合考评和完成率均为全省第一。

——突出保障和改善民生，社会建设全面加强。始终把维护群众利益作为政府工作的根本出发点，市、县两级财政用于民生的支出达到70亿元，占到一般预算支出的44%。五大社会保险面进一步扩大，城乡低保对象实现应保尽保，新增就业人数51333人，下岗失业人员再就业10788人，城镇登记失业率为3.1%。转移农村劳动力48600人。临汾新高中完成主体工程，562所学校122.48万平方米的中小学危房改造基本完成。小学、初中巩固率和高中毛入学率分别达到99.3%、99.4%和86.5%，高考二本以上达线人数9077人，比上年增加1450人，教育质量大幅提升。临汾新医院完成主体工程，市第四人民医院和古县人民医院改革试点工作扎实推进，县、乡、村三级卫生机构标准化建设深入实施，新农合参保人数达到298.13万人，参合率达93.47%。建设各类保障性住房5269套32.9万平方米。人民群众的上学难、看病难、住房难问题得到有效缓解。“爱树建”活动深入开展。第六次人口普查取得阶段性成果。食品药品、质量监督、计划生育、广播电视、国防人防、民族宗教、外事侨务、地方志和信访、审计、统计、档案、防震、老龄等各项工作都取得了新的进展。市政府向人民承诺的十件实事全部兑现。坚持把民主法治和政府自身建设作为做好各项工作的重要保障，自觉接受人大法律监督、工作监督和政协民主监督，认真执行人大决议，坚持重大事项向人大报告和向政协通报制度，办理人大代表建议150件，政协委员提案331件，办复率达到100%。认真听取各民主党派、工商联、无党派人士意见，提高决策科学化、民主化水平。大力推进政府职能转变，不断强化抓工作的力度，政府的公信力、执行力大幅度提升。进一步加强行政执法监督和普法教育，严厉打击各类刑事犯罪，“平安临汾”建设迈出新步伐。

各位代表，过去的一年，是我市在科学发展的道路上，迈出步伐最快、取得成绩最大、人民得到实惠最多的一年，也是我们的思路更加清晰、士气得到提振的重要一年。现在的临汾，焕发着干事创业的朝气，呈现出蒸蒸日上的局面。这些成绩的取得，是省委、省政府和市委正确领导的结果，是市人大、市政协监督支持的结果，是全市广大干部群众奋发进取、共同努力的结果。在此，我代表市政府，向全市人民，向人大代表、政协委员，向各民主党派、人民团体、工商联和社会各界人士，向驻临部队、武警官兵和驻临单位，表示崇高的敬意和衷心的感谢!在看到成绩的同时，我们也清醒地认识到存在的问题和差距。主要是：经济结构需要进一步调整，发展方式亟待进一步转变，产业链条仍需进一步延伸，产业上下游运行不够紧密，解决这些问题，既有现实紧迫性，也有长期复杂性，转型发展任务十分艰巨；城镇化水平比较低，城乡、区域发展不平衡的矛盾比较突出；节能减排、安全生产压力依然较大；教育、医疗、环境等事关民生的社会事业发展任务依然繁重；部分干部创新意识不强、眼界不宽、解决复杂问题的办法不多，抓工作的精力不够集中；等等。对此，我们将积极采取措施，认真加以解决。按照省委、省政府对我市转型跨越、先行发展的总体要求，根据《中共临汾市委关于制定国民经济和社会发展第十二个五年规划的建议》，市政府组织各级各部门，深入调查研究，广泛征求意见，圆满完成了《“十二五”规划纲要（草案）》的编制工作。规划纲要以转型跨越为主题，以转变发展方式为主线，从九个方面对“十二五”经济社会发展作了阐述，并提出努力建设新型工业大市、区域中心城市、中西部经济强市，力争“十二五”末经济总量翻一番的宏伟目标。我们将进一步细化、量化，制定出分年度工作计划，加大实施力度，确保“十二五”各项目标任务圆满完成。

二、2011年目标任务

　　2011年是“十二五”开局之年，也是临汾经济社会全面跨越发展的关键之年。我们既面临着重大机遇，也面临着严峻挑战。从有利条件看，世界经济继续恢复增长，国家中部崛起战略的实施，特别是我省“转型综改试验区”的设立，给我们提供了极大的机遇和空间。从不利因素看，国际形势复杂多变，不确定因素依然较多；国家对货币市场、房地产市场调控政策进一步趋紧；我市促增长、转方式、控通胀、保民生的任务更加艰巨。我们必须准确判断形势，保持清醒头脑，增强赶超意识，励精图治，奋发图强，在新的起点上开创科学发展的新局面。

今年政府工作的总体思路是：全面贯彻落实党的十七届五中全会、省委九届十一次全会和市委二届八次全会精神，紧紧抓住资源型经济转型综合配套改革试验区的重大机遇，坚持以科学发展为主题，以转变发展方式为主线，以转型跨越、先行发展为主旋律，优化区域布局，调整经济结构，加强“三农”工作，加快城镇化进程，保护生态环境，推进改革创新，强化社会管理，保障改善民生，努力打造新型工业大市、区域中心城市、中西部经济强市，建设经济繁荣、社会和谐、民生殷实、环境优美的新临汾。

今年，我市经济社会发展的指导性指标是：地区生产总值增长14%左右；全社会固定资产投资增长25%以上；社会消费品零售总额增长16%；海关进出口总额增长15%；财政总收入增长16%-18%；一般预算收入增长16%-18%；城镇居民人均可支配收入增长10%；农民人均纯收入增长10%以上；城镇登记失业率控制在4.2%以内；居民消费价格总水平涨幅控制在4%左右。

约束性指标是：万元GDP综合能耗下降3.5%左右；二氧化硫、化学需氧量排放量分别下降2.36%和2%；氮氧化物、氨氮排放量分别下降2.19%和1.83%；工业烟尘、粉尘排放量下降2%；万元工业增加值用水量下降5%。

实现上述目标，重点做好以下八个方面的工作：

　　（一）以建设“百公里汾河新型工业城镇走廊”为引领，着力加快区域协调发展

　　促进区域协调发展，是转型跨越的基本要求。我市沿汾区域人口集中、区位独特、资源赋存丰富、产业基础较好，呈现出快速发展的势头。必须立足市情，突出重点，加快建设纵贯临汾腹地，北起霍州，南至侯马，以汾河为中轴，以大运高速和霍侯一级公路中间地带为区间的“百公里汾河新型工业城镇走廊”，把这一区域打造成新型工业、高效农业、现代城镇、文化旅游、生态文明高度发达的示范区和引领区，带动东西两山全面振兴，形成“沿汾崛起、两翼齐飞”的新格局。今年，首先启动洪洞至襄汾段的规划和建设，一年起好步，打好基础，构起框架，五年形成规模。

坚持四化统筹，构筑区域优势。“百公里汾河新型工业城镇走廊”是一个新型经济社会综合体。要强化“一盘棋”的观念，统筹工业新型化、农业现代化、市域城镇化和城乡生态化发展规划，突出发挥工业化提供就业、保障供给，城镇化接纳人口、提供需求两大优势，推动工业化、城镇化协调共进，带动农业现代化、城乡生态化提升水平。一是打造新型工业引领区。按照“高端化、全循环”的要求，加快传统产业整合改造，拉长产业链条，发展循环经济；大力扶持新兴产业，发展高、精、尖产品，壮大企业实力，努力建设重要的煤化工、优质钢和先进制造业基地。出台《加快园区发展建设的指导意见》，建立园区管理机构和服务组织。聚集要素、聚集项目、聚集优势，突出建设好洪洞甘亭工业园、洪洞赵城煤焦化深加工园、襄汾河西焦化工业园和侯马电子工业园及侯马国际保税物流港五大龙头园区，引领全市40个产业园区快速发展，使其成为发达地区产业转移的先行承接地。二是打造现代城市精品区。以“一城三区”同城化发展为核心，建好中心城市，统筹发展重点集镇，加快农村改造撤并，促进农民向市民、农民工向产业工人转化，提高城镇化水平。三是打造高效农业示范区。以产业化为方向，大力发展现代农业、设施农业，建设一批高效园区和示范基地。四是打造文化旅游核心区。挖掘、整合根祖文化、黄河文化、晋文化等资源，加快景区开发，策划品牌旅游线路，建设中西部乃至全国重要的旅游目的地。

推进三项建设，改善基础设施。抓好汾河深度治理，按照“统一规划，分段治理”的原则，加快城区段汾河生态修复治理二期工程，并以此为标准，南北延伸，两岸拓展，今年形成雏形，明年完成全部治理任务。启动涝河、洰河生态治理修复工程，营造大水大绿的生态环境。抓好保障性住房建设，实施20亿元的保障性住房建设项目。抓好交通设施建设，力争临汾民航机场年底试航；临吉高速、临汾北环高速、京昆与青兰高速临汾联络线三条高速公路竣工通车；完成大运高速临汾北出口道路拓宽改造工程；开工建设吉县—河津高速公路、滨河东路尧都—洪洞、尧都—襄汾段工程；加快霍侯一级公路改造，其中对洪洞—襄汾段按城市道路标准进行改造；桃临公路改造完成路基；沁洪线大加山—洪洞段完成前期，力争年内开工；做好洪洞—侯马城际轻轨前期工作；支持大西高铁、山西中南部通道、张台铁路加快进度。规划建设一批走廊内城市道路主网、村镇路网和旅游通道，基本形成“高速公路、国省干线公路、农村公路、旅游公路四网并重，公路、铁路、航空立体交叉”的现代交通网络。同时，配套建设电站电网、信息通讯、广播电视、给水排水、污水垃圾处理等基础设施，为百公里汾河新型工业城镇走廊创造良好的硬环境。

各位代表，“百公里汾河新型工业城镇走廊”的规划建设，是我市未来五至十年发展的重大战略和任务。我们要举全市之力，集全民之智，把“百公里汾河新型工业城镇走廊”建成全国有影响、中西部最具活力的示范区和引领区！

　　（二）以推进工业新型化为目标，着力构建现代产业体系按照“传统产业循环化、优势产业规模化、支柱产业多元化、新兴产业高端化”的总体思路，坚持“大项目带动、大企业引领、大园区承载”，构建具有临汾特色的现代产业体系。

改造提升传统产业。以延长产业链条为主攻方向，以循环经济为发展模式，以大企业大集团为主要依托，实施煤、焦、冶、电四大产业改造提升工程。继续深化煤炭资源整合改造。在关死关实小矿井的基础上，加快推进新规划矿井的扩能改造和煤矿复工复产步伐，保证30%达到规模产能，原煤产量达到5000万吨，确保全市下游产业链条需求和工业经济正常运行，确保居民生活、学校医院及供气供热等民生用煤。加快焦化、冶金两大产业的重组整合和改造提升步伐，用好我省“综改试验区”政策，出台《焦化企业重组整合意见》和《冶金企业重组整合意见》，完成同世达257万吨、安泽永鑫120万吨、襄汾新腾达120万吨、洪洞浩溢120万吨等7个焦化项目的整合重组，建成山西三维2.5万吨叔碳酸乙烯酯、浮山中强3.2万吨活性炭、山西铭宇6000吨H-酸单钠盐、襄汾万鑫达20万吨甲醇、古县利达20万吨甲醇等煤化工项目。开工建设中煤集团在蒲县、尧都、襄汾的规划项目和保利集团裕丰公司、华电集团公司项目。加快推进山西焦煤与神华合作1500万吨焦化项目，8月份开工建设60万吨烯烃项目。以优质普碳钢、特种合金钢为重点，加快太钢重组中宇及临钢退城搬迁步伐，力争上半年开工，完成翼钢500万吨优特钢升级改造一期50%的工程量，打造曲沃千万吨和翼城500万吨钢铁基地。发展绿色煤电，完成霍州国电2×60万千瓦、侯马电厂2×30万千瓦及临汾中电投和大唐国际两个2×30万千瓦机组二期工程，推进安泽冀氏4×60万千瓦、翼城4×60万千瓦、古县鑫秀2×35万千瓦机组等项目前期工作，力争启动建设。

培育壮大战略性新兴产业。积极承接东部产业转移，把发展非煤、非资源型产业作为结构调整的重点领域，突出新装备、新材料、新能源、新医药，促进一批新项目落地生成和做大做强。壮大装备制造业，建设平阳重工制造园区，扩大新产品生产。加快晋源实业7万吨合金铸造、新兴际华60万吨铸件一期、华翔美的35万吨精密铸件、山西锻造厂20万吨精密锻件等项目建设。大力发展纳米材料、LED、钕铁硼等新型材料，抓好山西天极磁材1000吨高中档钕铁硼磁材、洪洞飞虹微纳米LED外延片和芯片等项目。积极推进生物质能、煤层气、风能、太阳能等新能源的开发利用，抓好吉县煤层气钻探压缩液化等项目。整合改造医药产业，保护现有品牌，组建医药集团，逐步实现规模化发展。

加快发展现代服务业。突出抓好现代物流和旅游产业。重点支持侯马方略保税物流中心做大做强，积极申报2.3平方公里出口加工区，加快总占地56平方公里的山西国际陆港综合保税园区建设。上半年，完成规划，全面启动征地拆迁、基础设施建设和招商引资工作，年内部分企业落地入驻，全力打造黄河金三角现代物流基地。同时积极支持铁路、公路、邮政、烟草、煤炭等重点物流企业和其他市场主体拓展领域，创新方式，提升物流业发展水平。按照我市《关于加快旅游产业发展的意见》，实施精品带动、“快旅慢游”战略。突出洪洞大槐树寻根祭祖园的龙头地位，完成景区外围、民俗村的改造扩建工程，并进行整体包装策划，形成以根祖文化为纽带的旅游聚力点，构建“一带五区一线十点”的旅游产业发展布局。重点建设中镇霍山旅游区、大槐树文化旅游区、尧文化旅游区、丁村民俗文化旅游区、晋文化旅游区，加快七里峪、广胜寺、丁村景点开发建设，完成尧陵二期，启动三期工程，建成晋国博物馆，打造汾河文化旅游带。以黄河壶口为核心，加快壶口景区、克难坡、人祖山、管头山等景点的修复开发，开工建设明清古街、唐庙和大型文化演艺厅等工程，形成黄河风情旅游精品线路。

同时，抓好十大景点、22个旅游招商项目的落地实施，打响“中华根·黄河魂·尧乡韵”旅游品牌。加快发展商贸、餐饮、金融、信息等服务业，规划建设一批大商场、大市场，提高三产在国民经济中的比重。

（三）以发展高效现代农业为重点，着力巩固和加强农业基础地位

　　“三农”工作是强基固本的大事。始终把“三农”工作放在政府工作的首位，以促进农业增效和农民增收为核心，狠抓生产保供给，大兴水利强基础，力促增收惠民生。

全面落实强农惠农政策，不断加大“三农”投入。坚持农业投入“三个优先”，安排支农资金15.4亿元，确保粮食直补、良种补贴、农机具购置补贴等十项强农惠农政策落实到位。出台以煤补农、以矿帮村、以企支农的鼓励政策，深入开展机关结对帮扶活动，加大工业反哺农业、城市支持农村力度。

切实加强水利设施建设，提高农业综合生产能力。积极争取上级水利资金和政策，投资10亿元，加快农田灌溉、应急水源、水保生态、河道治理、病险水库改造和小水库工程建设。实施吉县东城、乡宁谭坪、永和阁西垣、大宁割麦垣等四项沿黄提水工程和禹门口东扩、五马水库、西里水电站、清峪水库等四项应急水源工程建设。完成10项小型水库除险加固工程。抓好洪洞、曲沃国家小型水利工程重点县建设。实施涝河、洰河、芝河、昕水河、滏河5条河道治理工程。完成中低产田改造26万亩，水土流失治理面积40万亩，坡改梯2万亩，建设淤地坝40座。

实施产业化四大工程，推动现代农业高效发展。一是实施蔬菜自给工程。新发展设施蔬菜3万亩，蔬菜总面积达到90万亩，总产达到27亿公斤，建设“菜园子”，保证“菜篮子”，确保“十二五”末全市蔬菜总量自给。二是实施粮食高产创建工程。确保粮食面积稳定在700万亩以上，粮食总产保持在17亿公斤左右。三是实施现代农业示范基地建设工程。突出抓好曲沃磨盘岭、尧都尧王台、襄汾丁村白莲等十大现代农业示范园区和水果、干果、蔬菜、药材四个百万亩基地建设。今年，十大示范园区基本成形，全市新增水果５万亩、干果15万亩、蔬菜４万亩、药材3万亩。加快畜牧产业化发展，力争畜牧业产值达到36亿元以上。四是实施“393”龙头企业培育工程。完善“公司+园区（基地）+农民专业合作组织+农户”的产业化发展模式，扶持龙头企业做大做强，全市农产品加工龙头企业年销售收入达到38亿元以上，新增农民专业合作社400个。

搞好“两平台一通道”建设，促进农业增产农民增收。充分发挥政府的组织和主导作用，由政府直接出资，为农民提供技术、销售及金融等方面的服务。一是搭建技术服务平台。开展技术下乡、人才下乡活动，围绕产业化大规模培训农民，完成农业新技术培训15万人次，实现乡乡有服务组织，村村有技术人员。二是搭建专业销售平台。采取农商对接、农超对接、订单收购的形式，建设大型农副产品交易市场和销售中介组织，大力培育农村经纪人；提升隰县金梨、吉县苹果、永和红枣、浮山贡米等品牌形象，组织参加省内外农产品交易会、博览会，推广网络交易，提高市场占有率和整体竞争力。三是建立农民小额贷款通道。实行“农民贷款、政府贴息”的办法，加大对农民小额贷款的支持力度，扶持农民发展特色产业，确保农民人均纯收入增幅不低于城镇居民人均可支配收入增幅，“十二五”末农民人均纯收入翻一番。

实施新的“五个全覆盖”，加快新农村建设。扎实推进农村街巷硬化、农村便民连锁店、农村文化体育场所、中等职业教育免费、新型农村社会养老保险等新的“五个全覆盖”工程。制定两年完成计划，逐村逐户落实任务，确保按期完成。进一步加大扶贫开发力度，完成整村推进41个村，移民搬迁5200人。加快新农村集中连片发展，完成240个重点推进村任务，建成20个示范连片区。

（四）以建设百万人口中心城市为核心，着力加快市域城镇化进程

　　顺应城镇化加速发展、工业化加速提升的新形势，统筹工业化、城镇化协调共进，坚定不移地走新型城镇化道路，实施城乡建设三年大提升计划。科学编制和严格落实城镇体系规划。按照“一带两圈多点”的城镇发展格局，编制完成《临汾城镇化发展规划》、《尧（都）洪（洞）襄（汾）城镇群规划》、《汾河城区段生态治理修复二期工程详细规划》、《涝、洰河生态治理工程详细规划》、《环城水系景观控制性详细规划》、《河西新城城市设计》；完善《河西新城控制性详细规划》、《中心城区控制性详细规划》等六个控规和供水、供热及空港区等十个专项规划。认真做好12个大县城、60个重点镇的规划，把霍州、翼城、古县、乡宁建成工矿型县域中心，把安泽、浮山、汾西、蒲县、隰县、永和、大宁建成轻型工业主导型县域中心，把吉县建成旅游主导型县域中心。

加快城市基础设施建设。推进洪洞、尧都、襄汾“一城三区”同城化发展，市区建设新城、拉大框架，疏解老城、新旧融合，精心打造“一河两翼”核心区。按照分期开发、基础先行、滚动发展的思路，全面启动河西新城建设。完成西关大桥西延道路建设工程，开工建设高铁景观大道、河西南北大道、新高中南路、漪汾花园南路等四条道路工程。加快推进新高中、新医院、图书馆、博物馆、会展中心、奥体中心、广电中心、文化广场、高铁车站、城市规划展览馆等十大建筑，新高中在8月底投入运行，新医院年底完成门急诊楼的内装修，其余八大建筑开工建设。突出畅通、净化、增绿三大重点，加快旧城改造步伐。拓宽改造五一路中段，开工建设五一路东延工程，全面开通五一路通道。启动实施北内环立交桥和道路改造、二中路、北城壕防汛道路、古城公园二期以及中大街南段、西赵路西段等工程建设，并对城市主次街道拆违拆墙，大搞绿化，畅通道路，美化城市。同时，采取统一规划、市场运作的办法，加快城中村、城郊村改造。

切实加强城镇管理。按照“精心、精细、精品”的要求，强化街区管理职能，健全长效管理机制。集中力量整治交通秩序和市容市貌，完善数字化城市管理系统，大力开展爱国卫生运动，不断加强市民素质教育，巩固卫生城市创建成果。积极稳妥放宽户籍限制，着力解决好进城务工人员就业、安居、子女上学、社会保障、户籍管理等困难。

坚持以城带乡，城乡互动，推进城镇化进程，把城镇化建设和管理的理念、方法、公共设施投入等延伸到所有镇、村和居民集中居住区，让城乡居民共享城镇化成果。

（五）以低碳绿色发展为方向，着力建设两型社会

　　全面落实环保优先方针政策，突出抓好县（市、区）污水处理厂、垃圾处理场、工 业污染在线监测和城市集中供热供气等环保设施建设，力争实现“无空白县和企业”，所有企业废水全循环，废气达标排放，稳增二级天数，提高一级天数，建设资源节约型和环境友好型社会。

扎实抓好节能降耗。坚持源头控制、科技支撑、严格监管“三管齐下”，认真落实节能降耗目标责任制，做好合同能源管理、能耗限额管理等基础工作，建立节能预警调控机制，加强投资项目节能评估审查，大力推广节能技术，实施节能工程，强化节能执法，坚决淘汰落后产能，有效控制能源消费总量。

切实加强环境治理。围绕创建省级环保模范城市和提升全市环境质量两大目标，坚决打好打胜工业污染治理、城市环境综合整治、农村生态环境保护、水环境治理、蓝天碧水扩容提质五大硬仗。推进集中供热、供气体制改革，实施天然气接入工程，实现由煤气向天然气的转换；加快临汾中电、大唐国际热源厂投产运营，市区新增供热面积200万平方米。进一步加快各县(市、区）集中供热工程和垃圾处理场建设，实现“县县建成垃圾处理场”目标。市区在稳定二级以上天数的基础上，力争一级天数增加5%以上，大气综合污染指数稳定在1.8以下。

深入推进生态建设。实施市区生态体系建设和环城水系工程，打造北方山水城市。大力开展造林绿化，向荒山、荒坡、通道二次进军，重点推进昕水河流域百万亩生态经济型防护林体系和太行山水源涵养林建设，全年完成造林面积56万亩。各位代表，发展循环经济、减少环境污染、大搞生态建设、创建美好家园是时代的要求、人民的期盼。我们一定要采取更加有力的措施，把临汾建设成为环境优美、生态自然的宜居宜业魅力城市！

　　（六）以改革创新为动力，着力破解制约发展的难题

　　紧紧抓住“转型综改试验区”、煤炭可持续发展试点省、循环经济和生态试点省等政策机遇，进一步深化改革，创新机制，扩大开放，助推全市转型跨越发展。

深化十项改革。理顺国有资产监管体制，妥善解决5户国有企业改制遗留问题，积极推进其它市属企业改革，确保年内取得实质性突破。加快行政管理体制、乡镇机构改革，稳妥推进事业单位人事管理制度改革。深化城市管理体制改革，把绿化、市政、环卫等公共设施和服务推向市场，提高城市公共服务的质量和水平。深化农村土地流转综合配套改革，规范土地流转程序。

特别是探索建立跨县域统筹的方法和路子，推进区域一体化进程。在全面完成林权制度改革的基础上，积极推进造林补偿、林权转让和资源评估抵押等配套改革，基本建立现代林业产权制度。全面实施县域医药卫生一体化综合改革，完成医改五项重点任务。深化文化体制改革，推进经营性文化单位转企改制，扶持发展文化产业。深化粮食企业改革，盘活、用好现有资源，多渠道安置分流人员。深化金融改革，积极引进股份制商业银行，切实加强金融监管。深化财税改革，强化部门预算、国库集中支付、财税库银横向联网、非税收入收缴、政府采购，对政府投资的建设项目，实行风险评估和投资评审。同时，加快推进侯马城乡一体化改革、安泽新农村建设试点改革，做好全省“综改试验区”的相关工作。

拓宽筹资渠道。认真研究国家政策，积极争取政策性扶持。充分发挥投资公司作为城市建设投资和资本运营的主体作用，整合优质资产、改善股权结构、实施多元化资产运营，做强平台实力，实现平台转型和集团化实体运营，为公司上市做好准备。广泛加强与各金融投资机构的合作，利用发行城建债二期、中期票据、信托产品、短期融资债券等多种资本市场工具，争取融资50亿元。做好经营城市的大文章，盘活城市资源，规范土地一级市场，搞活二级市场，变资源为资本，筹集建设资金，推动城市发展。加强银企合作，争取晋商、光大等商业银行在我市设立分支机构，增设村镇银行，全年新增贷款500亿元。制定优惠和奖励政策，引导民间资本特别是煤炭资源整合后退出资金再投入，进行城市、新兴产业和基础设施建设。出台《关于加强招商引资工作的意见》，调动一切积极因素，大招商，招大商。加强与环渤海经济圈、长三角、珠三角互动，强化与黄河金三角、乌金三角经济区协作，积极组织和参加各类招商引资活动，争取一批世界500强和国内500强企业来我市投资办厂。全年力争通过各种渠道融通资金700亿元。

增加用地供给。积极争取上级用地指标，确保高于全省平均水平。围绕耕地占补平衡，实施3万亩造地计划，充分利用沟坝多造地、造好地，确保耕地总量不减少，质量不降低。抓住我省被国家确定为城乡用地增减挂钩试点省的机遇，力争我市3个县被列为全省试点县。加大建设用地复垦整合力度，增加财政投入，动员各方力量，实施万亩复垦整合计划，立足县域，全市统筹，整合利用，为重点项目建设提供用地支撑。

推进科技创新。把科技和人才作为转型跨越发展的重大战略，进一步加大科技平台和人才集聚平台的建设力度，加快企业技术中心建设，积极开展新产品开发和新技术推广，形成自主知识产权，提升企业的核心竞争力。加强与高校、科研院所的合作，完成临汾开发区和侯马开发区两个孵化器建设，促进科技创新成果转化应用。落实“柔性引才”政策，加大招才引智力度，建设“两高”人才队伍。发挥技工学校、职业技术学院作用，培育高技能实用型人才。利用农业产业化科技示范基地，创建科技示范乡、示范村、示范户，培养科技带头人。

发展民营经济。民营经济是县域经济的主要支撑。必须把民营经济作为壮大县域经济、增强发展活力的重要抓手，按照“一村一品、一县一业”的县域经济发展思路，充分发挥民营经济的机制优势，出台《支持民营经济发展的意见》，切实解决好项目审批、贷款担保、人才技术、用地供应等发展难题，积极引导民营企业加强与市外大企业、大集团联合，承接产业转移，引进项目、技术和资金，发展深加工、高新技术、现代服务业，壮大民营企业规模，激发县域经济发展的内生动力。

（七）以强化社会管理为抓手，着力建设平安和谐临汾

　　切实加强安全生产。安全问题是临汾的切肤之痛。必须以对人民高度负责，对临汾发展高度负责的态度，正确处理安全与发展的关系，坚持“安全第一、预防为主、综合治理”的方针，始终把安全工作放在各项工作的首位。继续深入开展“安全生产年”活动，进一步强化企业的主体责任。全面落实并严格执行各项安全生产制度。突出抓好煤矿、非煤矿山、尾矿库、冶金、道路交通、森林防火、食品药品、危险化学品、地质灾害、重点工程等行业和领域的安全工作。

扎实开展安全生产专项整治、打非专项行动和隐患排查治理。对证照齐全、合法经营、生产条件达标的，建立机制，加强监管；对证照不全、生产条件不达标的，彻底整顿，限期达标；对无证无照、违法非法生产的，坚决予以取缔，严格追究责任。同时，加大安全生产的教育培训力度，开工建设临汾安全生产职业技术学校，利用各种培训机构，加强对职工的安全教育，夯实安全基础，确保亿元GDP生产安全事故死亡率等六项相对指标控制在省控指标以内，逐步推进安全生产由明显好转向稳定好转转变。

扎实做好信访稳定工作。本着“社会要稳定、事情要解决”的原则，改进信访工作机制，落实信访工作责任，实行领导干部定期接访、定点包案和下访制度，妥善解决群众合理合法的诉求，有效化解社会矛盾，确保赴省集体上访和赴京非正常上访大幅度下降。加强社会治安综合治理，加快以信息化为支撑的社会管理创新工作，严厉打击“两抢一盗”等各类刑事犯罪，做好社区矫正工作，广泛开展普法教育，努力建设“平安临汾”。

进一步强化社会管理。规范劳动用工管理，维护劳动者合法权益。做好地震监测和防灾工作，提高防震减灾能力。加强应急管理，妥善处置各类突发事件。认真落实民族宗教政策，依法管理宗教事务。完善基层管理和服务体系，健全重大工程项目建设和重大政策制定的社会稳定风险评估机制。认真做好人口与计划生育工作，稳定低生育水平，提高出生人口素质。深入开展“爱树建”活动，大力弘扬“敢为人先、坚韧不拔、开放包容、勤劳智慧”的临汾精神。积极发展文化广电事业，完善公共文化体系，完成56个文化站、2445个农家书屋建设，启动20户以下已通电自然村“广播电视村村通”工程。发展以蒲剧、威风锣鼓为代表的非物质文化产业。广泛开展群众性文体活动，办好市第三届运动会。加强国防动员和民兵预备役建设，推进新形势下的双拥工作。继续做好外事侨务、档案、统计、地方志、妇女儿童、红十字、残疾人、老龄、人防、气象等各项工作。

（八）以改善民生为取向，着力提高人民群众生活质量

　　从解决人民群众最关心、最直接、最现实的利益问题入手，大力实施六大民生工程，办好十件惠民实事。

实施教育提质工程，确保学有所教。切实加大教育投入，逐步提高预算内教育经费占财政支出的比例。建好临汾新高中，确保秋季顺利招生。创建6个义务教育标准化县（市、区）、300所义务教育标准化学校。继续实施中小学布局调整和校舍改造提升工程，撤并百人以下学校134所，新建学校28所，改扩建学校48所，彻底消除危房。积极发展学前教育，完成市第二幼儿园和县（市、区）公办幼儿园建设任务，解决“入园难”问题。扩大中等职业教育规模。规范民办学校办学行为。启动山西师大的新校区建设。完成山西师大临汾学院的搬迁工作。新建襄汾、洪洞和改扩建尧都、曲沃、翼城的特教学校。支持临汾职业技术学院、临汾高级技工学校、临汾电大扩招发展。继续开展“三名”创建活动，进一步提高教师队伍素质。

实施医疗健康工程，确保病有所医。建好市人民医院，启动第四人民医院迁建工作，加快中医院和精神病医院的迁建前期准备。在全市所有政府办基层医疗卫生机构和村卫生室实施基本药物制度。进一步强化公共卫生体系和卫生执法体系建设，加强疾病预防控制、社区卫生服务和妇幼保健工作。加快农村卫生服务网络和卫生服务队伍建设，继续做好新型农村合作医疗工作，确保参合率稳定在90%以上。

实施就业创业工程，确保劳有所得。高度关注重点群体、困难群众就业问题。继续实施“三支一扶”、就业见习等扶持项目，努力拓展高校毕业生就业渠道。支持发展劳动密集型企业，增加就业岗位。健全完善面向全社会的职业技能培训制度，广泛开展“订单式”、“委托式”培训，促进各类人员就业再就业。全市城镇新增就业3.95万人、失业人员再就业9200人。

　　实施社会保障工程，确保老有所养。进一步扩大社会保险和城乡低保覆盖面，加强社保基金征缴和监管，努力实现应保尽保。认真落实基本养老保险关系跨地区转移接续办法。扩大国家和省级新农保试点县范围，力争两年内实现全覆盖。扎实做好被征地农民的社会保障工作。重视社会老龄化问题，关心关注老年人生活，做好市老年活动中心的前期准备工作。

实施住房安居工程，确保住有所居。加快保障性安居住房建设是新时期扩大内需、完善住房供应体系的重大举措。采取以商补公、租售并举、市场运作等方式，优先保障用地供应，统筹使用各类保障性住房建设资金，积极争取开行等金融机构贷款，鼓励社会资金参与建设和运营。加快廉租房、经济适用房、公共租赁房建设和棚户区、农村危房改造，配套搞好基础设施，改善居民的基本居住条件。

实施物价调控工程，确保市场稳定。全面加强价格调控和监管，保障主要农产品、基本生活必需品、重要生产资料市场供应。严厉打击食品药品、农资及其它领域的假冒伪劣、恶意炒作、串通涨价、哄抬物价等不法行为，维护良好的市场秩序。增发各类补贴，建立健全社会救助和保障标准与物价上涨挂钩的联动机制，努力克服物价上涨对低收入群体的影响。

各位代表，今年市政府将尽全力办好十件惠民实事：1.建成汾河生态文化公园，向市民开放。2.完成大运高速公路土门连接线工程，畅通临汾北出口。3.建成市区城西客运一级站，确保投入运营，方便群众出行。4.新建保障性住房3650套，31.4万平方米。5.解决3000名就业困难人员就业。6.对300名各类残疾儿童实施康复救助，为500户农村贫困残疾人实施危房改造。7.完成扶贫移民搬迁5200人，对全市6500户农村危房实施改造。8.发展农村体育事业，新建农民体育健身工程610个。9.建设和改造农村便民连锁商店2523个，其中新建928个，覆盖全市85%的行政村。10.对全市1500个行政村实施街巷硬化工程。各位代表，民为邦本，本固邦宁。六大工程、十件实事，与人民群众的生活息息相关。我们一定要不遗余力地把实事办好，好事办实，让人民群众共享改革发展的成果，过上更加幸福美满的生活！

　　三、加强政府自身建设

　　各位代表，临汾已站在一个新的历史起点上，面对繁重而艰巨的工作任务，我们将牢记宗旨，执政为民，更加努力地做好政府的各项工作。

解放思想，先行先试，建设创新型政府。教育和引导广大干部职工在更高层次、更高境界上推进思想解放，破除陈旧的发展观念、过时的思维定势和落后的行为习惯，研究新情况，领会新政策，运用新方法，积极探索建立推动经济发展、项目落地、招商引资、社会管理的政策措施和体制机制，大胆创新，先行先试，提高科学决策、协调统筹和解决疑难问题的能力。

强化服务，优化环境，建设效能型政府。制定出台财政、税收、土地等有利于外商投资的优惠政策，打造外来投资的“磁场”。深化机关效能建设，重点推进行政审批制度改革，加强政务中心建设，推行网上审批，严格落实首问负责制和限时办结制，特别是对重点项目的相关手续做到马上办、主动办、透明办、灵活办，坚持多服务、少干预，多帮忙、少添乱，努力营造让投资者进得来、留得住、做得大的最优发展环境。

依法行政，规范施政，建设法治型政府。健全政府议事规则，坚持依法决策、科学决策、民主决策。强化依法行政，依规办事、依律履责，确保政府工作经得起法律、历史和群众的检验。大力推进政务公开，增强政府工作透明度。自觉接受人大及其常委会的法律监督、工作监督和人民政协的民主监督，加强与各民主党派、工商联、无党派人士和人民团体的联系，认真办理人大代表、政协委员的议案、提案。充分发挥工青妇、科协、侨联等群团组织作用。支持人民法院、人民检察院依法履行职责。全面落实“六五”普法规划，加快法治临汾建设进程。努力扩大基层民主，保障人民群众的知情权、参与权、监督权。

改进作风，情系百姓，建设务实型政府。进一步加大抓落实的力度，明确目标责任，层层分解任务，强化督促检查，严格奖惩兑现，确保各项工作真正落到实处。进一步强化政府工作人员的宗旨意识，坚持深入基层，深入群众，尽心竭力为群众办实事、办好事，把工作开展在一线，问题解决在一线，着力解决好就业难、看病难、上学难以及征地拆迁、劳动争议等群众反映强烈的突出问题，让人民群众生活得更加安心、放心、舒心，对未来更有信心。

厉行节约，勤政廉政，建设廉洁型政府。认真落实党风廉政建设责任制和《廉政准则》，深入开展反腐倡廉教育。强化重点领域、关键环节的行政监察，加强财政资金、重大项目和民生工程的审计监督，开展煤焦、工程建设等领域突出问题的专项治理，坚决纠正损害群众利益的不正之风，严厉惩治腐败行为。大兴勤俭节约之风，坚决反对奢侈浪费，把有限的资金更多地用在发展经济、改善民生上，全力营造风清气正、干事创业的良好氛围。

各位代表，临汾正在崛起，前景催人奋进。客观形势鞭策着我们，人民期待激励着我们。让我们在中共临汾市委的坚强领导下，在市人大、市政协的监督支持下，倍加珍惜不可多得的历史机遇，倍加珍惜来之不易的发展势头，倍加珍惜团结一致、风清气正的大好局面，扎实工作，奋力拼搏，为推动转型跨越、先行发展，再造一个新临汾而努力奋斗！
